# Smoking and smokeless tobacco use in nine South and Southeast Asian countries: prevalence estimates and social determinants from Demographic and Health Surveys

**DOI:** 10.1186/s12963-014-0022-0

**Published:** 2014-08-28

**Authors:** Chandrashekhar T Sreeramareddy, Pranil Man Singh Pradhan, Imtiyaz Ali Mir, Shwe Sin

**Affiliations:** 1 Department of Population Medicine, Faculty of Medicine and Health Sciences, University Tunku Abdul Rahman, Bandar Sungai Long, Selangor, Malaysia; 2 Consultant Senior Public Health Officer (Influenza Surveillance Project), Patan Academy of Health Sciences, Lalitpur, Nepal; 3 Department of Physiotherapy, Faculty of Medicine and Health Sciences, University Tunku Abdul Rahman, Bandar Sungai Long, Selangor, Malaysia; 4 Department of Pre-clinical Sciences, Faculty of Medicine and Health Sciences, University Tunku Abdul Rahman, Bandar Sungai Long, Selangor, Malaysia

**Keywords:** Prevalence, Smoking, Smokeless tobacco use, Social determinants, South and Southeast Asia

## Abstract

**Background:**

In South and Southeast Asian countries, tobacco is consumed in diverse forms, and smoking among women is very low. We aimed to provide national estimates of prevalence and social determinants of smoking and smokeless tobacco use among men and women separately.

**Methods:**

Data from Demographic and Health Surveys completed in nine countries (India, Pakistan, Nepal, Bangladesh, Maldives, Philippines, Cambodia, Indonesia, and Timor Leste) were analyzed. Current smoking or smokeless tobacco use was assessed as response “yes” to one or more of three questions, such as “Do you currently smoke cigarettes?” Weighted country-level prevalence rates for socio-economic subgroups were calculated for smoking and smokeless tobacco use. Binary logistic regression analyses were done on STATA/IC (version 10) by ‘*svy’* command.

**Results:**

Prevalence and type of tobacco use among men and women varied across the countries and among socio-economic sub groups. Smoking prevalence was much lower in women than men in all countries. Smoking among men was very high in Indonesia, Maldives, and Bangladesh. Smokeless tobacco (mainly chewable) was used in diverse forms, particularly in India, among both men and women. Chewing tobacco was common in Nepal, Bangladesh, Maldives, and Cambodia. Both smoking and smokeless tobacco use were associated with higher age, lower education, and poverty, but their association with place of residence and marital status was not uniform between men and women across the countries.

**Conclusion:**

Policymakers should consider type of tobacco consumption and their differentials among various population subgroups to implement country-specific tobacco control policies and target the vulnerable groups. Smokeless tobacco use should also be prioritized in tobacco control efforts.

## Background

In 2010, globally, 54% of Disability adjusted Life Years (DALYS) were caused from non-communicable diseases (NCDs) [1] and tobacco smoking including second-hand smoke was one of the leading risk factors for global disease burden accounting for 6.3% of global DALYS [2]. If the current trend of tobacco use continues, it could cause approximately 8.3 million deaths annually by the year 2030 [3], and more than 80% of them may occur in low- and middle-income countries (LMICs) [4], where nearly two-thirds of the world’s smokers live [3]. In 2012, there were an estimated 967 million smokers from 187 countries [5], with the highest burden of tobacco use in high-income countries (HICs), intermediate in middle-income countries, and lowest in low-income countries. The deaths attributable to tobacco use are 18%, 11%, and 4% respectively [6]. Increasing rates of smoking in many LMICs and decreasing rates in HICs may lead to increased proportional tobacco-related mortality in LMICs [6],[7].

The Southeast Asia region is home to nearly 400 million tobacco users, who experience about 1.2 million deaths annually [7]. Although smokeless tobacco (SLT) use is common among women, smoking among women is increasing [8],[9]. In Southeast Asia, tobacco is used in diverse forms, including cigarettes or *bidis* (dried tobacco rolled in paper or leaf), SLT such as chewing *khaini* (tobacco with slaked lime and aromatic spices), *surti* (dried tobacco leaves for chewing), or *paan masala* (tobacco with aromatic spices), sucking *gutkha* (mixture of tobacco and molasses available in small sachets), applying *gul* or *gudaku* as dentifrice, and inhaling *nas and naswar* (nasal inhalation of tobacco powder) [10]. *Bidis* are popular in Bangladesh, India, Maldives, Nepal, and Sri Lanka, whereas *cheroots* are popular in Myanmar, and roll-your-own cigarettes (in palm leaves or paper) are popular in Thailand and Timor Leste. In Bangladesh, India, and Nepal, use of *gul, gudaku, mishri, masher, lal dantamanjan* as dentrifice, and *nas/naswar* is common [11]. SLT use in various forms is directly responsible for oropharyngeal cancers [12],[13].

Socio-economic differentials in tobacco use have existed in both developed [14],[15] and developing countries [16]-[18]. Studies have reported that tobacco consumption rates are higher in lower socioeconomic classes and less-educated groups [16],[17],[19]. Moreover, smoking prevalence is lower among women worldwide, particularly in South and Southeast Asia [20]. Therefore, assessing socio-economic differentials of tobacco use in Southeast Asia by population-based surveys will provide information about effectiveness of tobacco control measures and aid policymaking. The global tobacco surveillance system [21], World Health Organization (WHO) STEPS program [22], and WHO World Health Surveys (WHS) [23] have provided such information. However, these surveys cover several countries from various regions, but not all of the countries in a region. Moreover, the literature from these surveys has emphasized smoking and reported determinants of tobacco use [16],[17],[24], but not about SLT use, which is prevalent in South and Southeast Asia [11]. Demographic and Health Surveys (DHS) collect information about tobacco use in nationally representative samples of men and women and have provided national estimates of tobacco use for Nepal [25], India [26],[27], sub-Saharan Africa [18], and other countries [28]. We aimed to provide national estimates on prevalence and social determinants of tobacco smoking and smokeless tobacco use in South and Southeast Asian countries.

## Methods

### Data source

We used data from nationally representative samples of women and men from DHSs conducted between 2005–2006 and 2012–2013 in nine South and Southeast Asian countries: India, Pakistan, Nepal, Bangladesh, Maldives, Indonesia, Philippines, Cambodia, and Timor Leste (Table 1). DHS aims to provide reliable indicators about fertility, family planning, health, and nutrition of populations in developing nations [29],[30]. DHSs were implemented by country-level statistical offices or other local institutions and are technically supported by ORC (Opinion Research Corporation) Macro International Inc. of Calverton, Maryland, USA and financially supported by the United States Agency for International Development (USAID) [30].

**Table 1:** Summary table of survey details in each country included for analysis

**Country**	**Dates of fieldwork**	**Number of households selected**	**Number of women interviewed**	**Number of men interviewed**	**Overall response rates (%)**
India	November 2005 - August 2006	109041	124385	74369	92.4
Pakistan	October 2012 - April 2013	12943	13558	3134	89.9
Nepal	January 2011 - June 2011	10826	12674	4121	97.6
Bangladesh	March 2007 - August 2007	10400	10996^+^	3771	97.8
Maldives	January 2009 - October 2009	6443	7131	1727	77.0
Indonesia	May 2012 - July 2012	43852	45607	9306	95.0
Cambodia	July 2010 - January 2011	15667	18754	8239	96.5
Philippines	August 2008 - September 2008	12469	13594	*	97.7
Timor Leste	August 2009 - February 2010	11463	13137	4076	93.5

*Men were not interviewed in this country.

^+^Women were not asked about tobacco use in this country.

### Sampling and sample size

The final samples of households in DHSs were selected by two stages, stratified random sampling using population proportionate to size technique to include both rural and urban residents. Heads of each selected household answered general questions about the household and listed the household members. All women aged 15 to 49 years and men aged 15 to 49 years or more (up to 54 years in India, Indonesia, and Bangladesh and up to 64 years in Maldives) who were the usual residents were eligible to participate. Trained interviewers collected information about demographic and socio-economic factors and health status. Questions about tobacco use were asked of all eligible men and women [29],[30], except in Bangladesh and the Philippines (Table 1).

### Outcome variables

The following four identical questions were asked to elucidate information about tobacco use in all countries, but response options varied between countries (see below).

1) *Do you currently smoke cigarettes?* (response as ‘yes’ or ‘no’)

2) *In the last 24 hours, how many cigarettes did you smoke?* (response as numerical)

3) *Do you currently smoke or use any other type of tobacco?* (response as ‘yes’ or ‘no’)

4) *What (other) type of tobacco do you currently smoke or use?* (options provided were pipe, chewing tobacco, snuff, country-specific options, and others)

Additional country-specific options given were the use of hookah (sheesha), bidi, and cigars in Maldives; bidi in India, Pakistan, Nepal and Bangladesh; cigars in Philippines; hand-rolled tobacco in Timor Leste; *Pan Masala* and *Gutkha* in India; and nuswar in Pakistan. Each respondent was classified as ‘current smoker’ if the response to the first question was ‘yes,’ the response to the fourth question was ‘pipe,’ or if they were using hookah, bidi, cigars, or hand-rolled tobacco. The respondents were classified as ‘current SLT user’ if the response to the fourth question was any form of SLT, including ‘chewing’ tobacco, ‘Pan Masala’, ‘Gutkha,’ and ‘snuff.’

### Explanatory variables

Age, religion, and marital status were reclassified for logistic regression analyses. Religion was categorized as Hindu, Islam, Roman Catholic, Buddhist, and others. Each religion was classified into two categories, i.e., main religion of the country and others, except in India (Hindu, Muslim, and others). Age of the participant was recoded as 15–29, 30–39, and 40-49/≥40 years (for women and men, respectively). Marital status was classified as being married or single. Single constituted being never married, separated, or divorced. A cohabiting partner was included under single as its proportion was very small in most countries. In DHS, place of residence was classified as rural or urban; educational level was classified as ‘no education’, ‘primary’, ‘secondary,’ or ‘higher.’ Household wealth index, considered a reliable proxy for household economic status [31], was calculated based on a standard set of household assets, dwelling characteristics, and ownership of consumer items as observed by the interviewer. Participants were ranked on the basis of their household score by dividing them into quintiles where the first quintile was the poorest 20% of the households and the fifth quintile was the wealthiest 20% [32].

### Ethics statement

The institutional review boards of ORC Macro International Inc. and participating institutions in each country provided ethical clearance for DHSs. In each survey, participants were informed about voluntary participation and confidentiality of information and could refrain from responding to any of the questions. Before each interview, details of the survey were explained and informed consent was obtained. Written consent was not obtained since no intervention was applied to the participants.

### Data analysis

All analyses were done for men and women separately in each country. Descriptive analyses were done for smoking and SLT use. Overall weighted prevalence estimates for tobacco smoking and SLT use were calculated by including sample weights to account for the complex sampling design of DHS. Weighted prevalence estimates of smoking and SLT use were calculated according to age groups, religion, place of residence, marital status, education, and wealth quintiles. Binary logistic regression analyses were done to assess demographic (age was entered as continuous variable) and socio-economic factors associated with smoking and SLT use by SVY command on STATA/IC version 10 [33]. Beta-coefficients, their 95% confidence intervals, and p-values were calculated.

## Results

### Sample characteristics

The sample size of women interviewed was greater than for men since DHS mainly aimed to estimate indicators related to mother and child health (Table 1). In the 2008 Philippines DHS, men were not sampled, whereas in 2007 Bangladesh DHS, women were not asked about tobacco use. In Pakistan, Indonesia, and Maldives, information about religion was not collected (Table 2). Both men and women were mainly from rural areas in Maldives, Cambodia, Nepal, and Timor Leste, whereas in India, Pakistan, and Indonesia urban–rural distribution was nearly equal. More than half of men and women were aged between 15 and 29 years in all countries except Bangladesh, Maldives, and Indonesia, where about 45% of men were aged ≥40 years. Most participants were educated through primary and secondary level, except in the Philippines, where a third of women had received higher education. Nearly a third of men were uneducated in Bangladesh and Maldives. A high proportion of women (56.2% to 17.1%) were not educated in all countries except Indonesia (3.6%) and the Philippines (1.6%). Two-thirds or more of both men and women were married, but in Pakistan, Bangladesh, Maldives, and Indonesia ≥95% of men were married. Participants were evenly distributed across all wealth quintiles except in Maldives, Indonesia, and Timor Leste where the richest quintile was the smallest.

**Table 2:** Distribution of survey samples according to demographic and socioeconomic variables among men and women in South and Southeast Asian countries in Demographic and Health Surveys

**Variable**	**India Number (%) N = 74369**	**Pakistan Number (%) N = 3134**	**Nepal Number (%) N = 4121**	**Bangladesh Number (%) N = 3771**	**Maldives**^**‡**^**Number (%) N = 1727**	**Indonesia**^**‡**^**Number (%) N = 9306**	**Cambodia Number (%) N = 8239**	**Timor Leste Number (%) N = 4076**
**Men**								
Urban	38199 (51.4)	1521 (48.5)	1351 (32.8)	1443 (38.3)	274 (15.9)	4417 (47.5)	2606 (31.6)	1051 (24.9)
Rural	36170 (48.6)	1613 (51.5)	2770 (67.2)	2328 (61.7)	1453 (84.1)	4889 (52.5)	5633 (68.4)	3061 (75.1)
**Age group (years)**								
15-29	36595 (49.2)	750 (23.9)	2269 (55.1)	882 (23.4)	385 (22.3)	1630 (17.5)	4657 (56.5)	2227 (54.7)
30-39	18904 (25.4)	1224 (39.1)	1025 (24.8)	1196 (31.7)	522 (30.2)	3430 (36.9)	1836 (22.3)	981 (24.0)
≥40^**¶**^	18870 (25.4)	1160 (37.0)	827 (20.1)	1693 (44.9)	820 (47.5)	4246 (45.6)	1746 (21.2)	868 (21.3)
**Marital status**								
Currently married	4484 (60.4)	3085 (98.4)	2625 (63.7)	3734 (99.0)	1645 (95.3)	9260 (99.5)	4755 (57.7)	1993 (48.9)
Single*	29485 (39.6)	49 (1.6)	1493 (36.2)	37 (1.0)	82 (4.7)	--------------	3444 (41.8)	1924 (47.2)
Living with partner	--------------	-------------	3 (0.1)	--------------	---------------	46 (0.5)	40 (0.5)	159 (3.9)
**Educational level**								
No education	10696 (14.4)	849 (27.1)	498 (12.1)	1092 (29.0)	646 (37.4)	270 (2.9)	676 (8.2)	798 (19.6)
Primary	11474 (15.4)	536 (17.1)	815 (19.8)	1205 (32.0)	534 (30.9)	3185 (34.2)	3354 (40.7)	1070 (26.3)
Secondary	40745 (54.8)	1000 (31.9)	2139 (51.9)	944 (25.0)	428 (24.8)	4665 (50.1)	3666 (44.5)	2025 (49.7)
Higher	11423 (15.4)	749 (23.9)	669 (16.2)	530 (14.1)	119 (6.9)	1186 (12.7)	543 (6.6)	183 (4.5)
**Wealth index**								
Poorest	7085 (9.5)	584 (18.6)	711 (17.3)	595 (15.8)	351 (20.3)	2319 (24.9)	1412 (17.1)	791 (19.4)
Poorer	10278 (13.8)	581 (18.5)	688 (16.7)	718 (19.0)	413 (23.9)	1920 (20.6)	1420 (17.2)	824 (20.2)
Middle	14865 (20.0)	548 (17.5)	727 (17.6)	746 (19.8)	489 (28.3)	1786 (19.2)	1451 (17.6)	828 (20.3)
Richer	19346 (26.0)	641 (20.5)	861 (20.9)	739 (19.6)	284 (16.4)	1700 (18.3)	1661 (20.2)	876 (21.5)
Richest	22795 (30.7)	780 (24.9)	1134 (27.5)	973 (25.8)	190 (11.0)	1581 (17.0)	2295 (27.9)	757 (18.6)
**Religion**								
Hindu	54723 (73.6)	------------	3486 (84.6)	368 (9.8)	---------------	---------------	--------------	6 (0.1)
Islam	9583 (12.9)	------------	107 (2.6)	3380 (89.6)	---------------	---------------	123 (1.5)	5 (0.1)
Buddhist	1138 (1.5)	------------	352 (8.5)	9 (0.2)	---------------	---------------	7812 (94.8)	0 (0)
Roman Catholic	6651 (8.9)	------------	80 (1.9)	5 (0.1)	----------------	---------------	59 (0.7)	4006 (98.3)
Others	2260 (3.0)	------------	96 (2.3)	0 (0)	---------------	---------------	243 (2.9)	59 (1.5)
Missing	14 (0.0)	------------	------------	9 (0.2)	---------------	---------------	--------------	--------------
	**India Number (%) N = 124385**	**Pakistan Number (%) N = 13558**	**Nepal Number (%) N = 12674**	**Maldives**^**‡**^**Number (%) N = 7131**	**Indonesia**^**‡**^**Number (%) N = 45607**	**Cambodia Number (%) N = 18754**	**Philippines Number (%) N = 13594**	**Timor Leste Number (%) N = 13137**
**Women**								
Urban	56961 (45.8)	6351 (46.8)	3701 (29.2)	1041 (14.6)	22898 (50.2)	6077 (32.4)	6762 (49.7)	3233 (24.6)
Rural	67424 (54.20)	7207 (53.2)	8973 (70.8)	6090 (85.4)	22709 (49.8)	12677 (67.6)	6832 (50.3)	9904 (75.4)
**Age group (years)**								
15-29	67415 (54.2)	5338 (39.4)	7200 (56.8)	3038 (42.6)	20956 (45.9)	10296 (54.9)	6976 (51.3)	7443 (56.7)
30-39	34025 (27.4)	4738 (34.9)	3258 (25.7)	2353 (33.0)	13745 (30.2)	4173 (22.2)	3644 (26.8)	3200 (24.4)
40-49	22945 (18.4)	3482 (25.7)	2216 (17.5)	1740 (24.4)	10906 (23.9)	4285 (22.9)	2974 (21.9)	2494 (18.9)
**Marital status**								
Currently married	87925 (70.7)	13010 (96.0)	9459 (74.6)	6558 (92.0)	32361 (71.0)	11439 (61.0)	7071 (52.0)	7548 (57.5)
Single*	36460 (29.3)	548 (4.0)	3214 (25.4)	573 (8.0)	12901 (28.2)	7218 (38.6)	5030 (37.0)	5260 (40.0)
Living with partner	----------	-----------	1 (0.0)	--------------	345 (0.8)	97 (0.5)	1493 (11.0)	329 (2.5)
**Educational level**								
No education	39769 (32.0)	7625 (56.2)	4877 (38.5)	1941 (27.2)	1622 (3.6)	3203 (17.1)	218 (1.6)	3922 (29.9)
Primary	17756 (14.3)	1831 (13.5)	2149 (17.0)	2503 (35.1)	13732 (30.1)	8796 (46.9)	2840 (20.9)	3112 (23.7)
Secondary	53882 (43.3)	2415 (17.8)	4584 (36.2)	2384 (33.4)	23759 (52.1)	6141 (32.7)	6267 (46.1)	5804 (44.2)
Higher	12966 (10.4)	1687 (12.4)	1064 (8.4)	303 (4.2)	6494 (14.2)	614 (3.3)	4269 (31.4)	299 (2.3)
**Wealth index**								
Poorest	14077 (11.3)	2486 (18.3)	2446 (19.3)	1578 (22.1)	10642 (23.3)	3260 (17.4)	2562 (18.8)	2544 (19.4)
Poorer	17652 (14.2)	2586 (19.1)	2296 (18.1)	1850 (25.9)	9187 (20.1)	3159 (16.8)	2664 (19.6)	2562 (19.5)
Middle	23682 (19.0)	2589 (19.1)	2336 (18.4)	1931 (27.1)	8678 (19.0)	3242 (17.3)	2648 (19.5)	2715 (20.7)
Richer	30136 (24.2)	2657 (19.6)	2516 (19.9)	1112 (15.6)	8478 (18.6)	3735 (19.9)	2771 (20.4)	2820 (21.5)
Richest	38838 (31.2)	3240 (23.9)	3080 (24.3)	660 (9.3)	8622 (18.9)	5358 (28.6)	2949 (21.7)	2496 (19.0)
**Religion**								
Hindu	89957 (72.3)	------------	10829 (85.4)	--------------	--------------	-------------	--------------	18 (0.1)
Islam	16742 (13.5)	------------	331 (2.6)	--------------	--------------	312 (1.7)	887 (6.5)	20 (0.2)
Buddhist	1765 (1.4)	------------	1058 (8.3)	--------------	--------------	17799 (94.9)	0 (0.0)	0 (0.0)
Roman Catholic	10977 (8.8)	------------	236 (1.9)	--------------	--------------	111 (0.6)	10453 (76.9)	12833 (97.7)
Others	4786 (3.9)	------------	220 (1.7)	--------------	--------------	528 (2.8)	2254 (16.6)	266 (2.0)
Missing	158 (0.1)	------------	--------------	--------------	---------------	4 (0.0)	--------------	-------------

^‡^Information about religion was not collected in these countries. *Includes never married, separated and divorced.

^¶^Up to 54 years in India, Indonesia, and Bangladesh, and up to 64 years in Maldives.

### Prevalence of smoking and SLT use

**Figure 1:**
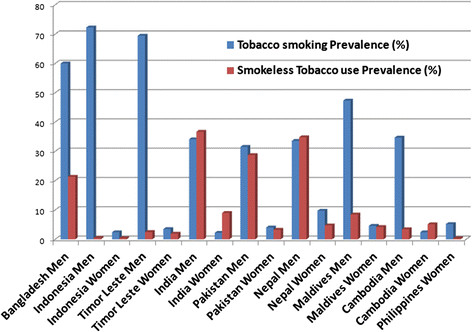
Prevalence of smoking and SLT use among men and women in nine South and Southeast Asian countries.

**Figure 2:**
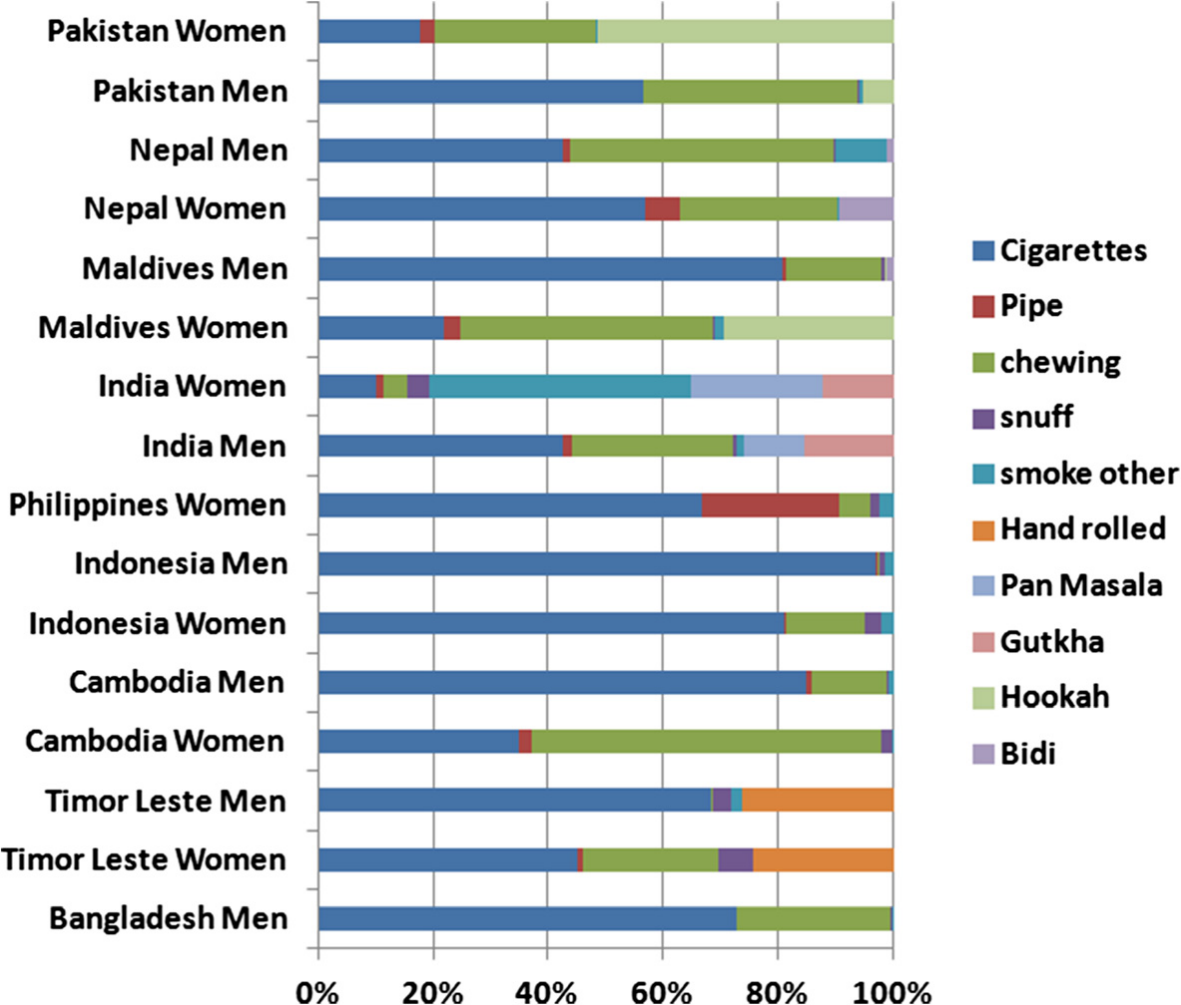
Proportional distribution of various forms of tobacco consumed among men and women in nine South and Southeast Asian countries.

Among men, weighted prevalence of smoking varied between the countries; the highest prevalence was found in Indonesia (72.3%), followed by Timor Leste (69.5%), Bangladesh (60.0%), and Maldives (47.3%), but prevalence was lower in India (34.1%), Nepal (33.6%), Cambodia (34.7%), and Pakistan (31.6%). Prevalence of SLT use among men also varied between countries, with the highest prevalence in India (36.7%), followed by Nepal (34.8%) and Bangladesh (21.4%), and the lowest in Indonesia (0.46%) and Timor Leste (2.5%) (Figure 1). Among women, weighted prevalence of smoking was much lower than men in all countries; the highest prevalence was in Nepal (9.8%), followed by Philippines (5.2%), Maldives (4.6%), and Pakistan (4.02%). Prevalence of SLT use among women was highest in India (9.0%), followed by Cambodia (5.1%), Nepal (4.8%), and Maldives (4.2%) (Figure 1). The most common form of tobacco consumed was cigarettes in all countries except India and Timor Leste. Cigarettes/bidis were smoked in India, and hand-rolled cigarettes were smoked in Timor Leste. However, Indian men and women used diverse forms of SLT including ‘*gutkha,’ ‘pan masala,’* and other chewing tobacco (unspecified). Chewing tobacco was also common in Nepal, Bangladesh, and Cambodia. Indonesian men mostly smoked cigarettes, while women there were also using chewing tobacco. Smoking a pipe/cigar was only seen among Filipino and Nepalese women, while women from Maldives and Pakistan mostly smoked hookah (Figure 2).

### Prevalence of smoking and SLT use according to socio-economic and demographic factors

Differentials in smoking and SLT use according to socio-economic and demographic variables are shown separately for men and women in Tables 3 and 4, respectively. Prevalence of both smoking and SLT use among men was higher in rural areas than urban areas in all countries (for example, among Indian men 36.8 vs. 29.5 and 39.9 vs. 31.3, respectively). Smoking and SLT use among women was higher in rural areas in India, Pakistan, Nepal, and Cambodia, but these differentials varied for prevalence of smoking and SLT use among women in other countries. Prevalence of smoking and SLT use among both men and women varied according to wealth quintiles and educational level. In all countries among men and women, prevalence of smoking and SLT use was generally highest among the least educated and the lowest among those with higher education. Prevalence of tobacco use was highest among the poorest and lowest among the richest. For example, among Nepalese men, prevalence rates for smoking and SLT use in those with no education vs. the highest education was 57.9 vs. 20.5 and 57.8 vs. 18.4, respectively. Among Bangladeshi men prevalence rates for smoking and SLT use in the poorest group vs. the richest was 70.1 vs. 46.8 and 26.6 vs. 15.7, respectively. The only exception was seen in Maldivian men, where differentials were very small for prevalence of smoking (poorest vs. richest was 47.6 vs. 52.9) (Table 3). Prevalence of both smoking and SLT use was higher among married men and women in all countries except the Philippines, Pakistan, Indonesia, and Maldives. Prevalence of both smoking and SLT use was higher among men and women aged ≥40 years in all countries, except for smoking among men and women in Maldives and SLT use among Pakistani and Indonesian men. Among Indian, Pakistani, Nepalese, and Maldivian women differentials by age were very high. For example, among Maldivian women, prevalence of smoking and SLT use in the 15–29 age group vs. those 40–49 years old was 1.1 vs. 10.4 and 0.2 vs.10.6, respectively (Table 4).

**Table 3:** Weighted prevalence rates (and 95% CIs) for tobacco smoking and smokeless tobacco use among men according to demographic and socio-economic variables in seven South and Southeast Asian countries in Demographic and Health Surveys

	**Tobacco smoking (%)**							
	**India**	**Pakistan**	**Nepal**	**Bangladesh**	**Maldives**^**‡**^	**Indonesia**^**‡**^	**Cambodia**	**Timor Leste**
**Overall prevalence (%)**	**34.12 (33.64, 34.6)**	**31.55 (28.78, 34.31)**	**33.59 (31.23, 35.95)**	**60.01 (58.18, 61.84)**	**47.33 (44.16, 50.49)**	**72.30 (70.85, 73.75)**	**34.69 (33.16, 36.22)**	**69.49 (67.64, 71.25)**
Urban	29.48 (28.79, 30.16)	27.95 (22.95, 32.96)	27.68 (24.64, 30.72)	54.77 (50.77, 58.78)	46.87 (40.32, 53.42)	69.05 (66.80, 71.29)	21.89 (19.21, 24.57)	63.99 (60.44, 67.54)
Rural	36.79 (36.15, 37.43)	33.51 (30.21, 36.81)	34.83 (32.05, 37.61)	61.55 (59.48, 63.62)	47.59 (44.33, 50.84)	75.63 (73.87, 77.40)	38.01 (36.25, 39.76)	71.46 (69.38, 73.53)
**Age group (years)**								
15-29	24.31 (23.68, 24.93)	21.15 (16.85, 25.45)	27.44 (24.72, 30.15)	56.94 (53.71, 60.18)	50.08 (43.67, 56.49)	77.27 (74.28, 80.27)	20.20 (18.55, 21.86)	60.76 (58.27, 63.26)
30-39	40.57 (39.60, 41.54)	30.45 (26.14, 34.76)	36.38 (31.73, 41.03)	57.68 (54.48, 60.88)	49.63 (44.08, 55.19)	73.16 (70.96, 75.36)	50.16 (47.04, 53.28)	80.15 (77.34, 82.95)
≥40^¶^	45.88 (44.90, 46.86)	39.94 (35.07, 44.82)	46.61 (42.01, 51.21)	63.27 (60.52, 66.01)	44.47 (40.15, 48.79)	69.91 (67.91, 71.90)	56.78 (53.77, 59.79)	79.47 (76.10, 82.85)
**Marital status**								
Married	41.64 (41.02, 42.27)	31.07 (28.25, 33.90)	40.52 (37.57, 43.48)	60.03 (58.17, 61.88)	46.42 (43.08, 49.77)	72.31 (70.86, 73.76)	48.47 (46.46, 50.47)	80.24 (78.06, 82.43)
Single*	20.52 (19.86, 21.19)	54.57 (37.57, 71.56)	21.43 (18.82, 24.04)	58.38 (39.87, 76.89)	62.91 (49.64, 76.18)	64.97 (41.88, 88.06)	15.30 (13.61, 16.98)	59.14 (56.66, 61.63)
**Education**								
No education	52.06 (50.82, 53.30)	40.41 (35.94, 44.87)	57.90 (52.58, 63.21)	72.33 (69.40, 75.26)	46.25 (41.65, 50.85)	72.32 (64.10, 80.55)	66.90 (62.12, 71.68)	81.42 (78.28, 84.57)
primary	44.87 (43.64, 46.10)	33.36 (27.75, 38.97)	42.83 (38.26, 47.40)	63.59 (60.58, 66.59)	52.40 (47.29, 57.51)	78.14 (75.94, 80.33)	46.17 (44.09, 48.26)	76.56 (73.72, 79.39)
Secondary	27.56 (26.95, 28.17)	28.92 (24.34, 33.49)	27.39 (24.37, 30.41)	51.88 (47.72, 56.04)	48.59 (42.38, 54.81)	73.12 (71.20, 75.04)	22.79 (20.95, 24.63)	62.03 (59.49, 64.56)
Higher	20.12 (19.00, 21.24)	18.56 (13.54, 23.57)	20.54 (16.71, 24.37)	35.63 (30.22, 41.04)	29.67 (20.39, 38.95)	50.86 (45.88, 55.85)	4.51 (2.67, 6.34)	60.60 (52.34, 68.85)
**Wealth index**								
Poorest	44.98 (43.61, 46.36)	32.86 (28.28, 37.45)	42.55 (36.92, 48.18)	70.12 (66.00, 74.23)	52.98 (46.13, 59.83)	80.98 (78.69, 83. 28)	52.40 (48.88, 55.93)	73.36 (69.81 (76.92)
Poorer	41.53 (40.30, 42.75)	35.22 (29.64, 40.80)	37.43 (33.11, 41.75)	63.97 (59.62, 68.32)	48.55 (42.52, 54.59)	78.80 (76.28, 81.31)	42.43 (39.21, 45.66)	73.09 (69.60, 76.58)
Middle	36.73 (35.67, 37.79)	36.51 (31.24, 41.79)	37.84 (32.31, 43.37)	63.18 (58.89, 67.47)	45.84 (40.71, 50.97)	76.62 (73.88, 79.35)	34.99 (31.91, 38.07)	71.81 (68.13, 75.48)
Richer	30.31 (29.37, 31.24)	32.64 (25.55, 39.73)	29.07 (24.29, 33.85)	56.43 (52.13, 60.74)	43.03 (35.14, 50.93)	68.11 (64.88, 71.34)	29.26 (26.67, 31.84)	69.19 (65.69, 72.69)
Richest	22.50 (21.70, 23.31)	21.9 (16.40, 27.39)	26.54 (23.17, 29.90)	46.81 (42.71, 50.91)	47.56 (39.32, 55.79)	57.91 (54.20, 61.62)	19.47 (17.03, 21.91)	61.54 (57.73, 65.35)
**Religion**								
Hindu	34.30 (33.77, 34.83)	--------------	34.19 (31.68, 36.70)	---------------------	--------------------	--------------------	---------------------	----------------------
Islam	37.69 (36.28, 39.09)	--------------	---------------------	61.65 (53.54, 69.77)	--------------------	--------------------	---------------------	-----------------------
Buddhist	--------------------	--------------	--------------------	--------------------	--------------------	--------------------	34.22 (32.69, 35.76)	--------------------
Roman catholic	--------------------	--------------	---------------------	---------------------	--------------------	--------------------	----------------------	69.78 (67.96, 71.59)
Others	23.56 (22.12, 25.00)	--------------	30.38 (25.56, 35.20)	59.83 (57.99, 61.68)	--------------------	--------------------	51.71 (42.92, 60.50)	51.02 (34.21, 67.82)
	**Smokeless tobacco use (%)**							
	**India**	**Pakistan**	**Nepal**	**Bangladesh**	**Maldives**^**‡**^	**Indonesia**^**‡**^	**Cambodia**	**Timor Leste**
**Overall prevalence (%)**	**36.72 (36.23, 37.21)**	**16.30 (14.16, 18.43)**	**34.82 (32.4, 37.24)**	**21.35 (19.48, 23.22)**	**8.48 (7.02, 9.94)**	**0.46 (0.28, 0.64)**	**3.43 (2.88, 3.98)**	**2.48 (1.8, 3.15)**
Urban	31.26 (30.56, 31.96)	20.13 (16.08, 24.17)	26.86 (23.22, 30.49)	20.19 (17.68, 22.71)	6.07 (3.34, 8.79)	0.13 (0.01, 0.25)	1.30 (0.62, 1.97)	1.86 (0.65, 3.08)
Rural	39.87 (39.21, 40.52)	14.21 (11.76, 16.66)	36.49 (33.67, 39.32)	22.53 (20.49, 24.57)	9.87 (8.16, 11.57)	0.78 (0.45, 1.12)	3.97 (3.30, 4.64)	2.69 (1.89, 3.50)
**Age group (years)**								
15-29	33.81 (33.11, 34.50)	21.04 (16.78, 25.30)	22.31 (19.50, 25.13)	14.30 (11.84, 16.76)	1.51 (−0.22, 3.25)	0.46 (0.15, 0.77)	1.96 (1.43, 2.50)	1.82 (1.16, 2.49)
30-39	42.03 (41.05, 43.02)	16.07 (12.84, 19.30)	50.86 (46.64, 55.07)	17. 90 (15.38, 20.43)	4.92 (2.81, 7.02)	0.37 (0.12, 0.61)	4.78 (3.72, 5.84)	3.11 (1.69, 4.53)
≥40	36.90 (35.92, 37.87)	13.27 (10.62, 15.93)	48.40 (43.88, 52.91)	28.09 (25.69, 30.49)	14.11 (11.38, 16.83)	0.52 (0.28, 0.76)	5.86 (4.66, 7.05)	3.40 (2.17, 4.63)
**Marital status**								
Married	41.38 (40.75, 42.01)	16.49 (14.33, 18.65)	47.28 (44.45, 50.11)	21.66 (20.09, 23.23)	8.72 (7.15, 10.29)	0.45 (0.27, 0.63)	4.72 (3.99, 5.45)	3.16 (2.20, 4.12)
Single*	28.30 (27.55, 29.06)	6.84 (1.12, 1.26)	12.97 (10.48, 15.46)	18.91 (6.22, 31.62)	3.94 (0.33, 7.55)	1.92 (−0.38, 4.22)	1.60 (1.11, 2.08)	1.81 (1.18, 2.45)
**Education**								
No education	45.58 (44.33, 46.83)	18.36 (14.45, 22.28)	57.79 (50.64, 64.95)	24.84 (22.09, 27.59)	17.21 (13.79, 20.63)	1.91 (−0.13, 3.96)	8.66 (6.00, 11.31)	4.17 (2.61, 5.73)
primary	44.25 (43.01,45.48)	19.77 (15.29, 24.26)	49.97 (45.83, 54.11)	25.25 (22.46, 28.04)	6.76 (3.67, 9.85)	0.79 (0.40, 1.18)	5.12 (4.16, 6.09)	2.70 (1.63, 3.77)
Secondary	34.39 (33.74, 35.05)	15.01 (11.83, 18.19)	27.71 (25.01, 30.41)	18.13 (15.45, 20.81)	1.96 (0.38, 3.54)	0.21 (0.10, 0.32)	1.42 (1.03, 1.81)	1.74 (1.11, 2.38)
Higher	22.97 (21.78, 24.15)	10.67 (7.27, 14.06)	18.39 (14.35, 22.44)	13.04 (9.91, 16.18)	4.31 (−1.18, 9.80)	0.03 (−0.03, 0.10)	0.08 (−0.03, 0.19)	1.93 (−0.43, 4.30)
**Wealth index**								
Poorest	51.51 (50.13, 52.90)	19.24 (14.08, 24.40)	42.90 (36.70, 49.09)	26.60 (23.18, 30.02)	11.13 (7.54, 14.71)	1.71 (0.98, 2.44)	7.21 (5.50, 8.92)	3.24 (1.90, 4.58)
Poorer	45.19 (43.95, 46.43)	16.50 (12.53, 20.47)	40.45 (35.34, 45.57)	25.38 (21.86, 28.90)	11.66 (7.86, 15.45)	0.25 (0.06, 0.43)	5.41 (3.93, 6.89)	3.61 (1.75, 5.48)
Middle	37.28 (36.19, 38.36)	14.69 (10.57, 18.80)	44.28 (38.14, 50.42)	24.13 (20.94, 27.31)	8.72 (5.82, 11.62)	0.38 (−0.00, 0.78)	3.34 (2.19, 4.48)	2.08 (1.13, 3.02)
Richer	32.52 (31.56, 33.49)	14.25 (10.52, 17.98)	29.87 (25.92, 33.82)	19.35 (16.26, 22.44)	6.83 (3.60, 10.07)	0.03 (−0.03, 0.09)	1.42 (0.93, 1.91)	2.32 (1.05, 3.59)
Richest	23.81 (22.97, 24.65)	17.00 (11.27, 22.74)	23.42 (19.31, 27.52)	15.65 (13.06, 18.25)	5.64 (2.24, 9.05)	0.10 (−0.01, 0.22)	0.79 (0.32, 1.25)	1.38 (0.41, 2.35)
**Religion**^**+**^								
Hindu	37.52 (36.97, 38.07)	--------------	35.53 (32.90, 38.15)	----------------------	----------------------	----------------------	----------------------	----------------------
Islam	35.97 (34.53, 37.40)	--------------	---------------------	---------------------	---------------------	---------------------	----------------------	-----------------------
Buddhist	--------------------	--------------	--------------------	--------------------	---------------------	--------------------	3.28 (2.72, 3.85)	-----------------------
Roman catholic	---------------------	--------------	----------------------	----------------------	----------------------	----------------------	-------------------	2.41 (1.74, 3.08)
Others	26.74 (25.16, 28.32)	--------------	31.02 (25.76, 36.27)	----------------------	----------------------	----------------------	8.55 (2.31, 14.78)	5.75 (0.34, 11.16)

^‡^Information about religion was not collected in these countries.

*Includes never married, separated, and divorced.

^+^For all countries except India (Hindu, Islam, and others), religion was grouped as main religion and others (for example Buddhist in Cambodia and Islam in Maldives, Indonesia, and Bangladesh).

^¶^Up to 54 years in India, Indonesia, and Bangladesh and up to 64 years in Maldives.

**Table 4:** Weighted prevalence rates (and 95% CIs) for tobacco smoking and smokeless tobacco use among women according to demographic and socio-economic variables in seven South and Southeast Asian countries in Demographic and Health Surveys

	**Tobacco smoking (%)**							
	**India**	**Pakistan**	**Nepal**	**Philippines**	**Maldives**^**‡**^	**Indonesia**^**‡**^	**Cambodia**	**Timor Leste**
**Overall prevalence (%)**	**2.2 (2.08, 2.31)**	**4.02 (3.28, 4.77)**	**9.75 (8.7, 10.8)**	**5.22 (4.81, 5.63)**	**4.6 (3.94, 5.25)**	**2.39 (2.15, 2.64)**	**2.41 (1.76, 3.07)**	**3.49 (3.07, 3.91)**
Urban	0.81 (0.72, 0.91)	1.85 (1.08, 2.62)	4.83 (3.79, 5.88)	5.47 (4.89, 6.06)	4.58 (3.29, 5.88)	2.47 (2.10, 2.83)	0.63 (0.40, 0.85)	4.01 (2.93, 5.08)
Rural	2.87 (2.71, 3.03)	5.12 (4.09, 6.14)	10.57 (9.36, 11.78)	4.89 (4.34, 5.44)	4.61 (3.86, 5.35)	2.31 (1.99, 2.63)	2.88 (2.06, 3.70)	3.30 (2.87, 3.73)
**Age group (years)**								
15-29	0.95 (0.86, 1.05)	1.61 (1.07, 2.17)	2.78 (2.20, 3.35)	3.86 (3.34, 4.37)	1.09 (0.52, 1.66)	1.47 (1.20, 1.75)	0.95 (0.49, 1.42)	1.44 (1.11, 1.77)
30-39	3.07 (2.81, 3.32)	4.29 (3.31, 5.27)	13.80 (11.92, 15.67)	5.54 (4.74, 6.34)	4.69 (3.48, 5.89)	2.49 (2.06, 2.91)	3.30 (2.48, 4.12)	4.74 (3.88, 5.61)
40-49	4.62 (4.24, 5.00)	7.53 (5.89, 9.16)	26.11 (23.17, 29.05)	8.04 (7.02, 9.05)	10.4 (8.74, 12.08)	3.86 (3.32, 4.40)	4.92 (3.28, 6.56)	7.85 (6.65, 9.04)
**Marital status**								
Married	2.60 (2.46, 2.74)	3.83 (3.13, 4.54)	11.77 (10.49, 13.05)	4.79 (4.13, 5.45)	3.91 (3.29, 4.53)	2.48 (2.21, 2.76)	2.89 (2.22, 3.56)	4.63 (4.03, 5.23)
Single*	1.00 (0.86, 1.14)	7.93 (3.95, 11.93)	3.41 (2.63, 4.19)	5.48 (4.96, 5.99)	11.69 (7.84, 15.54)	2.13 (1.75, 2.52)	1.65 (0.92, 2.38)	1.92 (1.52, 2.32)
**Education**								
No education	4.37 (4.12, 4.62)	6.49 (5.35, 7.65)	20.18 (17.78, 22.58)	14.49 (9.63, 19.34)	10.16 (8.42, 11.91)	8.14 (5.46, 10.82)	8.60 (6.39, 10.82)	6.21 (5.32, 7.11)
primary	1.64 (1.41, 1.87)	1.27 (0.56, 1.99)	7.52 (5.97, 9.08)	8.08 (6.97, 9.20)	4.68 (3.53, 5.84)	2.59 (2.21, 2.96)	2.08 (1.44, 2.72)	3.92 (3.16, 4.69)
Secondary	0.44 (0.37, 0.51)	0.50 (0.18, 0.84)	1.09 (0.69, 1.48)	4.70 (4.12, 5.27)	1.22 (0.63, 1.82)	2.10 (1.80, 2.41)	0.06 (−0.0, 0.12	1.60 (1.22, 1.98)
Higher	0.23 (0.10, 0.35)	0.28 (0.04, 0.59)	0.27 (−0.02, 0.56)	3.89 (3.27, 4.50)	2.74 (−1.71, 7.21)	1.51 (0.95, 2.07)	---------------------	1.62 (0.09, 3.15)
**Wealth index**								
Poorest	4.91 (4.50, 5.32)	8.77 (6.65, 10.88)	23.75 (20.90, 26.59)	6.93 (5.90, 7.95)	6.55 (5.05, 8.06)	3.89 (3.18, 4.61)	5.99 (4.09, 7.89)	4.10 (3.26, 4.95)
Poorer	3.45 (3.11, 3.79)	5.47 (3.94, 7.01)	12.56 (10.51, 14.61)	5.77 (4.76, 6.78)	5.18 (3.88, 6.48)	2.39 (1.88, 2.90)	3.64 (2.30, 4.98)	4.23 (3.28, 5.17)
Middle	1.97 (1.75, 2.19)	3.94 (2.89, 4.98)	7.74 (6.41, 9.07)	5.32 (4.33, 6.31)	3.89 (2.80, 4.98)	1.92 (1.51, 2.33)	2.02 (1.42, 2.62)	3.42 (2.63, 4.21)
Richer	0.92 (0.78, 1.07)	2.01 (1.07, 2.94)	5.56 (4.29, 6.84)	4.21 (3.44, 4.98)	3.73 (2.29, 5.18)	2.32 (1.76, 2.89)	1.01 (0.61, 1.41)	2.79 (2.11, 3.47)
Richest	0.42 (0.33, 0.50)	0.37 (0.13, 0.61)	2.78 (1.88, 3.67)	4.52 (3.72, 5.31)	3.89 (2.23, 5.55)	1.72 (1.28, 2.17)	0.21 (0.07, 0.35)	3.09 (2.11, 4.06)
**Religion**^**+**^								
Hindu	2.28 (2.15, 2.40)	----------------	9.46 (8.34, 10.58)	--------------------	----------------------	--------------------	---------------------	---------------------
Islam	2.27 (1.93, 2.60)	----------------	--------------------	-------------------	-----------------------	--------------------	---------------------	--------------------
Buddhist	--------------------	----------------	--------------------	--------------------	--------------------	--------------------	2.14 (1.48, 2.80)	---------------------
Roman catholic	------------------	----------------	--------------------	5.50 (5.02, 5.97)	--------------------	---------------------	--------------------	3.50 (3.07, 3.93)
Others	0.93 (0.74, 1.12)	----------------	11.28 (8.86, 13.70)	4.11 (3.39, 4.83)	---------------------	---------------------	12.14 (6.94, 17.34)	3.02 (1.03, 5.01)
	**Smokeless tobacco use (%)**							
	**India**	**Pakistan**	**Nepal**	**Philippines**	**Maldives**^**‡**^	**Indonesia**^**‡**^	**Cambodia**	**Timor Leste**
**Overall prevalence (%)**	**9.0 (8.8, 9.21)**	**2.44 (1.94, 2.96)**	**4.75 (3.8, 5.68)**	**0.32 (0.23, 0.41)**	**4.23 (3.46, 5.0)**	**0.41 (0.29, 0.52)**	**5.13 (4.52, 5.75)**	**1.93 (1.65, 2.2)**
Urban	5.99 (5.71, 6.27)	2.07 (1.42, 2.72)	2.46 (1.60, 3.33)	0.12 (0.05, 0.20)	2.49 (0.99, 3.98)	0.16 (0.07, 0.24)	0.72 (0.35, 1.09)	1.65 (1.07, 2.23)
Rural	10.47 (10.20, 10.74)	2.64 (1.95, 3.33)	5.12 (4.03, 6.20)	0.55 (0.38, 0.73)	5.10 (4.19, 5.99)	0.67 (0.45, 0.88)	6.30 (5.53, 7.07)	2.02 (1.71, 2.33)
**Age group (years)**								
15-29	5.19 (4.98, 5.40)	1.42 (0.89, 1.94)	2.03 (1.47, 2.59)	0.08 (0.02, 0.14)	0.16 (0.31, 0.96)	0.29 (0.16, 0.42)	0.86 (0.55, 1.17)	0.41 (0.26, 0.57)
30-39	12.42 (11.97, 12.88)	2.35 (1.69, 3.00)	6.48 (5.01, 7.96)	0.26 (0.11, 0.41)	4.01 (2.87, 5.14)	0.42 (0.27, 0.56)	5.82 (4.64, 7.01)	2.17 (1.66, 2.68)
40-49	15.33 (14.72, 15.93)	4.26 (3.11, 5.40)	10.82 (8.51, 13.12)	0.94 (0.60, 1.27)	10.63 (8.77, 12.50)	0.57 (0.40, 0.75)	14.33(12.67, 15.99)	6.00 (4.92, 7.07)
**Marital status**								
Married	10.22 (9.97, 10.48)	2.36 (1.85, 2.87)	5.81 (4.66, 6.96)	0.09 (0.00, 0.17)	4.17 (3.39, 4.95)	0.41 (0.29, 0.54)	6.49 (5.61, 7.36)	2.66 (2.24, 3.07)
Single*	5.37 (5.06, 5.68)	4.33 (2.39, 6.28)	1.36 (0.84, 1.87)	0.46 (0.32, 0.59)	4.80 (2.70, 6.91)	0.38 (0.23, 0.52)	2.97 (2.48, 3.46)	0.92 (0.66, 1.17)
**Education**								
No education	14.49 (14.09, 14.90)	3.56 (2.76, 4.36)	7.94 (6.29, 9.59)	5.66 (2.69, 8.64)	11.61 (9.63, 13.59)	2.26 (1.09, 3.44)	12.91 (10.94,14.87)	4.37 (3.65, 5.08)
Primary	10.63 (10.07, 11.20)	1.52 (0.83, 2.22)	6.13 (4.18, 8.07)	0.72 (0.42, 1.02)	4.04 (2.42, 5.67)	0.52 (0.36, 0.68)	5.78 (5.04, 6.52)	1.84 (1.33, 2.35)
Secondary	3.95 (3.73, 4.16)	0.93 (0.49, 1.38)	1.42 (0.97, 1.86)	0.16 (0.07, 0.25)	0.20 (0.00, 0.40)	0.29 (0.16, 0.42)	0.72 (0.44, 1.0)	0.49 (0.30, 0.69)
Higher	1.10 (0.87, 1.33)	0.10 (0.08, 0.27)	0.16 (−0.15, 0.47)	0.09 (−0.01, 0.19)	0.77 (−0.33, 1.88)	0.03 (−0.01, 0.08)	-------------------	--------------------
**Wealth index**								
Poorest	17.81 (17.14, 18.49)	4.64 (2.97, 6.31)	9.67 (6.27, 13.07)	1.19 (0.78, 1.60)	7.85 (6.04, 9.67)	1.29 (0.84, 1.73)	11.14 (9.53, 12.75)	3.66 (2.78, 4.55)
Poorer	11.99 (11.44, 12.53)	2.40 (1.50, 3.30)	6.22 (4.54, 7.90)	0.47 (0.21, 0.73)	5.54 (4.16, 6.93)	0.46 (0.21, 0.71)	7.46 (6.22, 8.71)	1.89 (1.33, 2.45)
Middle	8.60 (8.16, 9.04)	2.13 (1.29, 2.96)	4.29 (3.09, 5.49)	0.11 (−0.00, 0.24)	3.29 (2.30, 4.28)	0.28 (0.13, 0.43)	5.76 (4.76, 6.76)	1.80 (1.31, 2.30)
Richer	5.89 (5.54, 6.25)	2.34 (1.61, 3.07)	3.52 (2.47, 4.58)	0.10 (−0.04, 0.24)	2.74 (1.55, 3.91)	0.08 (−0.00, 0.17)	2.31 (1.54, 3.07)	1.56 (1.06, 2.07)
Richest	2.88 (2.64, 3.11)	0.88 (0.48, 1.29)	1.37 (0.91, 1.83)	---------------------	2.23 (−0.11, 4.59)	0.09 (0.00, 0.18)	0.60 (0.30, 0.90)	1.05 (0.60, 1.51)
**Religion**^**+**^								
Hindu	8.96 (8.73, 9.18)	---------------	4.37 (3.37, 5.36)	---------------------	---------------------	--------------------	---------------------	---------------------
Islam	9.34 (8.76, 9.92)	---------------	--------------------	---------------------	--------------------	--------------------	---------------------	---------------------
Buddhist	---------------------	---------------	---------------------	---------------------	---------------------	---------------------	5.03 (4.41, 5.64)	
Roman catholic	------------------	---------------	---------------------	0.27 (0.17, 0.37)	---------------------	--------------------	---------------------	1.94 (1.66, 2.22)
Others	8.86 (8.19, 9.53)	---------------	6.70 (4.58, 8.81)	0.48 (0.26, 0.70)	---------------------	--------------------	8.78 (3.25, 14.31)	1.08 (0.08, 2.07)

^‡^Information about religion was not collected in these countries.

*Includes never married, separated, and divorced.

^+^For all countries except India (Hindu, Islam, and others), religion was grouped as main religion and others (for example Buddhist in Cambodia and Islam in Maldives, Indonesia, and Bangladesh).

### Association of tobacco use with socio-economic and demographic factors

The association of tobacco use among men and women with socio-economic and demographic factors was assessed using binary logistic regression analysis. Men from rural areas had greater likelihood of smoking and SLT use in India only. Women from rural areas had greater likelihood of smoking in all countries except Nepal and Cambodia and SLT use in India, Cambodia, and Timor Leste (Table 5). Older men were more likely to smoke and use SLT in most countries (except for SLT use in Indonesia and Timor Leste). Older men were less likely to smoke in Maldives (β = −0.03) and Indonesia (β = −0.01).Older women were more likely to smoke and use SLT in all countries (β coefficients ranged from 0.04 to 0.12, Table 5). Married men were more likely to smoke and use SLT in all countries except Bangladesh (for both smoking and SLT use), Maldives, Indonesia, Cambodia, and Timor Leste (for SLT use only). Married women were more likely to smoke in the Philippines but were less likely to smoke in Indonesia and Maldives. Married women were more likely to use SLT in Nepal only (Table 5). Smoking and SLT use among both men and women were strongly associated with education (protective effect) in all countries, except for SLT use among men from Bangladesh and Timor Leste. Individuals who were educated were less likely to smoke or use SLT. Smoking and SLT use among men was associated with wealth in all countries except Nepal, Pakistan, Timor Leste, and Maldives, while the association of wealth with smoking and SLT use among women was seen in all countries except Timor Leste (for smoking). Wealthier individuals were less likely to smoke or use SLT. Smoking among men was associated with religion in India, Nepal, Cambodia, and Timor Leste, while SLT use among men was associated with religion in India and Nepal. Smoking among women was associated with religion in India, Philippines, and Cambodia, while SLT use among women was associated with religion in India and Nepal (data about religion was not collected in Indonesia and Maldives).

**Table 5:** **Binary logistic regression analyses for demographic and socio-economic factors associated with tobacco smoking and smokeless tobacco use among men and women of nine South and** ***Southeast Asian*** **countries in Demographic and Health Surveys**

	**Tobacco smoking (men)**															
	**India (β, 95 % CIs)**	**p-value**	**Pakistan**	**p-value**	**Nepal (β, 95% CIs)**	**p-value**	**Bangladesh (β, 95% CIs)**	**p-value**	**Maldives**^**‡**^**(β, 95% CIs)**	**p-value**	**Indonesia**^**‡**^**(β, 95 % CIs)**	**p-value**	**Cambodia (β, 95% CIs)**	**p-value**	**Timor Leste (β, 95% CIs)**	**p-value**
**Urban/Rural**	−0.11 (−0.16, −0.05)	<0.001	0.15 (−0.16, 0.46)	0.334	0.08 (−0.17, 0.33)	0.541	−0.02 (−0.23, 0.19)	0.852	−0.36 (−0.77, 0.06)	0.092	−0.09 (−0.25, 0.06)	0.225	−0.05 (−0.25, 0.16)	<0.647	0.12 (−0.10, 0.34)	0.290
**Age (years)**	0.03 (0.03, 0.03)	<0.001	0.04 (0.02, 0.05)	<0.001	0.02 (0.01, 0.03)	<0.001	0.01 (−0.00, 0.02)	0.066	−0.03 (−0.04, −0.01)	0.001	−0.01 (−0.02, −0.01)	<0.001	0.07(0.06, 0.08)	<0.001	0.06 (0.05, 0.08)	<0.001
**Marital status**	−0.41 (−0.48, −0.34)	<0.001	0.83 (0.10, 1.57)	0.027	−0.38 (−0.62, −0.14)	0.002	−0.06 (−0.95, 0.82)	0.891	0.69 (0.08, 1.30)	0.027	−0.96 (−1.67, −0.26)	0.008	−0.56 (−0.76, −0.36)	<0.001	−0.14 (−0.33, 0.05)	0.158
**Education**	−0.34 (−0.37, −0.31)	<0.001	−0.27 (−0.39, −0.14)	<0.001	−0.47 (−0.58, −0.36)	<0.001	−0.44 (−0.55, −0.33)	<0.001	−0.28 (−0.44, −0.12)	0.001	−0.32 (−0.43, −0.21)	<0.001	−0.69 (−0.80,-0.58)	<0.001	−0.32 (−0.43, −0.20)	<0.001
**Wealth index**	−0.15 (−0.18, −0.13)	<0.001	−0.02 (−0.13, 0.10)	0.767	−0.03 (−0.12, 0.05)	0.423	−0.09 (−0.17, −0.02)	0.019	−0.10 (−0.22, 0.03)	0.121	−0.23 (−0.29, −0.17)	<0.001	−0.25 (−0.31, −0.19)	<0.001	−0.06 (−0.12, 0.01)	0.092
**Religion**^**+**^	−0.10 (−0.14, −0.07)	<0.001	---------	--------	−0.30 (−0.55, −0.05)	0.019	0.17 (−0.17, 0.51)	0.325	---------	--------	-----------	---------	0.73 (0.25, 1.21)	<0.003	−0.98 (−1.78, −0.18)	0.016
	**Smokeless tobacco use (men)**															
**Urban/Rural**	−0.14 (−0.19, −0.09)	<0.001	−0.82 (−1.15, −0.49)	<0.001	0.12 (−0.14, 0.38)	0.355	−0.06 (−0.32, 0.20)	0.657	−0.08 (−0.77, 0.61)	0.815	0.77(0.03, 1.51)	0.042	−0.07 (−0.72, 0.58)	<0.833	−0.02 (−0.75, 0.71)	0.956
**Age (years)**	−0.00(−0.01, −0.00)	0.005	−0.03 (−0.05, −0.01)	0.001	0.03 (0.02, 0.04)	<0.001	0.04 (0.03, 0.05)	<0.001	0.05 (0.03, 0.08)	<0.001	0.02 (−0.02, 0.06)	0.335	0.04 (0.03, 0.06)	<0.001	0.02 (−0.01, 0.05)	0.272
**Marital status**	−0.55 (−0.62, −0.49)	<0.001	−1.10 (−2.08, −0.13)	0.027	−1.19 (−1.48, −0.91)	<0.001	−0.28 (−1.24, 0.68)	0.562	−0.77 (−1.75, 0.20)	0.12	0.25 (−1.11, 1.62)	0.717	−0.15 (−0.46, 0.15)	<0.330	−0.27 (−0.86, 0.33)	0.381
**Education**	−0.07 (−0.10, −0.04)	<0.001	−0.21 (−0.36, −0.06)	0.006	−0.41 (−0.56, −0.26)	<0.001	−0.09 (−0.19, 0.02)	0.107	−0.50 (−0.94, −0.07)	0.024	−0.63 (−1.12, −0.14)	0.012	−0.50 (−0.69, −0.30)	<0.001	−0.25 (−0.50, 0.01)	0.055
**Wealth index**	−0.28 (−0.30, −0.26)	<0.001	−0.14 (−0.28, 0.00)	0.054	−0.10 (−0.19, −0.00)	0.041	−0.16 (−0.24, −0.08)	<0.001	−0.09 (−0.28, 0.09)	0.328	−0.69 (−1.13, −0.25)	0.002	−0.44 (−0.60, −0.28)	<0.001	−0.16 (−0.32, 0.01)	0.063
**Religion**^**+**^	−0.14 (−0.17, −0.10)	<0.001	----------	---------	−0.35 (−0.64, −0.06)	0.017	−0.15 (−0.55, 0.24)	0.438	---------	---------	---------	---------	0.82 (−0.02, 1.67)	<0.057	0.90 (−0.07, 1.87)	0.068
	**Tobacco smoking (women)**															
	**India**		**Pakistan**		**Nepal**		**Philippines**		**Maldives**^**‡**^		**Indonesia**^**‡**^		**Cambodia**		**Timor Leste**	
**Urban/Rural**	0.36 (0.21, 0.51)	<0.001	−0.15 (−0.68, 0.37)	0.566	−0.24 (−0.56, 0.07)	0.125	−0.39 (−0.57, −0.21)	<0.001	−0.82 (−1.35, −0.30)	0.002	−0.41 (−0.61, −0.21)	<0.001	0.06 (−0.43, 0.54)	<0.821	−0.60 (−0.93, −0.27)	<0.001
**Age (years)**	0.07 (0.06, 0.07)	<0.001	0.07 (0.05, 0.09)	<0.001	0.10 (0.09, 0.11)	<0.001	0.04 (0.03, 0.05)	<0.001	0.08 (0.05, 0.10)	<0.001	0.05 (0.04, 0.06)	<0.001	0.06 (0.05, 0.07)	<0.001	0.07 (0.05, 0.08)	<0.001
**Marital status**	−0.16 (−0.32, −0.00)	0.050	0.45(−0.11, 1.00)	0.116	−0.21 (−0.47, 0.06)	0.129	−0.40 (−0.62, −0.19)	<0.001	0.30 (0.15, 0.46)	<0.001	0.35 (0.12, 0.58)	0.003	0.00 (−0.32, 0.32)	<0.996	−0.06 (−0.32, 0.21)	0.674
**Education**	−0.60 (−0.69, −0.52)	<0.001	−0.81(−1.07, −0.56)	<0.001	−0.67 (−0.82, −0.52)	<0.001	−0.37 (−0.49, −0.25)	<0.001	−0.40 (−0.76, −0.03)	0.032	−0.16 (−0.34, 0.02)	0.075	−1.17 (−1.39, −0.95)	<0.001	−0.37 (−0.52, −0.21)	<0.001
**Wealthindex**	−0.38 (−0.43, −0.33)	<0.001	−0.49(−0.64, −0.34	<0.001	−0.53 (−0.63, −0.43)	<0.001	−0.12 (−0.19, −0.05)	0.001	−0.28 (−0.43, −0.12)	0.001	−0.22 (−0.30, −0.15)	<0.001	−0.50 (−0.63, −0.38)	<0.001	−0.08 (−0.17, 0.02)	0.115
**Religion**^**+**^	−0.10 (−0.20, −0.00)	0.042	--------	--------	0.15 (−0.14, 0.44	0.307	−0.40 (−0.61, −0.19)	<0.001	---------	---------	---------	---------	1.63 (1.08, 2.18)	<0.001	−0.13 (−0.79, 0.53)	<0.704
	**Smokeless tobacco use (women)**															
**Urban/Rural**	−0.21 (−0.27, −0.14)	<0.001	−0.63 (−1.09, −0.18)	0.007	−0.01(−0.44, 0.43)	0.968	0.19 (−0.57, 0.95)	0.628	−0.34 (−0.98, 0.31)	<0.306	0.57 (−0.03, 1.18)	0.064	1.07 (0.49, 1.65)	<0.001	−0.48 (−0.86, −0.10)	0.013
**Age (years)**	0.06 (0.05, 0.06)	<0.001	0.05 (0.03, 0.07)	<0.001	0.07 (0.06, 0.08)	<0.001	0.10 (0.06, 0.14)	<0.001	0.10 (0.06, 0.14)	<0.001	0.04 (0.01, 0.06)	0.006	0.12 (0.11, 0.13)	<0.001	0.10 (0.08, 0.11)	0.000
**Marital status**	−0.05 (−0.12, 0.02)	0.192	0.33 (−0.17, 0.84)	0.195	−0.72 (−1.10, −0.34)	<0.001	0.22 (−0.75, 1.19)	0.652	−0.08 (−0.25, 0.09)	<0.364	0.44 (−0.02, 0.90)	0.061	−0.19 (−0.40, 0.01)	<0.067	0.03 (−0.29, 0.36)	0.841
**Education**	−0.28 (−0.32, −0.24)	<0.001	−0.60 (−0.83, −0.36)	<0.001	−0.19 (−0.36, −0.01)	<0.040	−0.62 (−1.16, −0.08)	0.023	−0.54 (−1.06, −0.03)	<0.040	−0.37 (−0.83, 0.09)	0.115	−0.48 (−0.64, −0.31)	<0.001	−0.54 (−0.77, −0.31)	<0.001
**Wealthindex**	−0.42 (−0.45, −0.39)	<0.001	−0.31 (−0.46, −0.16)	<0.001	−0.39 (−0.55, −0.24)	<0.001	−0.78 (−1.11, −0.45)	<0.001	−0.34 (−0.52, −0.16)	<0.001	−0.61 (−0.80, −0.42)	<0.001	−0.48 (−0.57, −0.39)	<0.001	−0.20 (−0.32, −0.08)	0.001
**Religion**^**+**^	0.13 (0.08, 0.17)	<0.001	------------	--------	0.45 (0.03, 0.86)	0.037	−0.08 (−0.67, 0.52)	0.803	---------	---------	---------	---------	0.54 (−0.26, 1.34)	<0.188	−0.63 (−1.60, 0.34)	0.203

^‡^Information about religion was not collected in these countries.

^+^For all countries except India (Hindu, Islam, and others), religion was grouped as main religion and others (for example Buddhist in Cambodia and Islam in Maldives, Indonesia, and Bangladesh).

## Discussion

Our report using DHS datasets provided national-level estimates and information about the pattern of tobacco use in nine countries in the South and Southeast Asia region. Our disaggregated analyses by gender and type of tobacco use demonstrated that pattern of tobacco consumption has cross-country and intracountry variations. In each country, tobacco consumption among men and women was unequally distributed in all demographic and socio-economic groups. Tobacco use among women was very low in all countries, but smoking was higher in Nepal and SLT use higher in India than other countries. Prevalence of smoking and SLT use among men was almost equal in India and Nepal, but among Bangladeshi men, smoking was higher than SLT use. Prevalence of smoking among men was very high in Indonesia, Timor Leste, and Maldives while SLT use was very low. In all countries, significant associations between age, education, and wealth for both smoking and SLT use highlights the existence of social disparities in tobacco use.

Prevalence estimates were comparable to DHS-based estimates for India [26] and Nepal [25] and were much higher than estimates for India and Nepal in GATS [17],[34], but prevalence in Cambodia was lower compared to another national survey [35]. Only three (India, Bangladesh, and the Philippines) of the nine countries that had also participated in the first wave of GATS did not allow comparison of prevalence in all GATS countries [17],[34]. Moreover, our estimates cannot be compared with those of GATS and WHS, which defined current smoking as smoking of any form of tobacco either daily or occasionally [16],[34], while the Global Burden of Disease (GBD) study defined daily smoking as smoking any type of tobacco product at least once per day [5]. Our estimates for the current smoking rate among men in Indonesia and Timor Leste were approximately 70%, whereas GBD reported rates as <61%. Similarly, among Nepalese women, our estimate for current smoking was 10%, but GBD results put daily smoking rate at 17%. Differences as large as these between our estimates and those from GBD may have occurred due to differences in data sources, definition of smoking, and statistical methods used for estimating prevalence rates [5]. GBD used multiple sources of microdata and aggregate data from major multicountry survey programs such as DHS, GATS, WHS, WHO STEPwise Approach to Surveillance program, and others, national-level, multiyear surveys on health, addiction, and risk factors, and three large health databases. To estimate prevalence of daily smoking, GBD analyzed data from the sources listed above for 2,102 country-years from 181 countries for a 33-year period, while we used cross-sectional samples of men and women from one DHS in each of the nine South and Southeast Asian countries for 2005–2013. GBD defined a daily smoker as someone who smokes any type of tobacco product at least once per day, but we defined current smoker as someone who responded as “yes” to the question, “Do you currently smoke cigarette?”. Moreover, GBD adopted robust statistical techniques such as regression analyses to adjust for data providing varying or non-standard definitions for smoking, but we relied on participants’ response to three questions to define current smokers. GBD estimates are more realistic, as the study used a spatial-temporal regression model and Gaussian process regression to create a complete time-series for all data from multiple sources, followed by computation of age-standardized prevalence rates, while our study computed the weighted point prevalence of current smoking among men and women, which was not age-standardized. Such methodological heterogeneity of tobacco surveys and the importance of more systematic design of surveys, questionnaires, and definitions have been previously underscored [25],[26]. Comparison with DHS-based reports for India and Nepal shows an increase in prevalence of tobacco use [25],[26]. Low prevalence of smoking and SLT use among women as reported earlier [17],[34] is not surprising since it is socially unacceptable for women to smoke in South Asian communities [36]. However, SLT use was common in India, Nepal, and Cambodia, confirming the results of previous studies [11],[37].

Our findings that current smoking is prevalent from the age of 15 years onwards but was higher in older age groups which is similar to results of previous surveys [16]-[18],[34]. This may be due to a cohort effect (i.e., smoking was less likely to be initiated in more recent decades). This means that more attention should be paid to young men in Indonesia, Maldives, and Bangladesh, where prevalence rates of smoking were alarmingly high. Some think that tobacco companies have been aggressively marketing to young people in these countries, particularly in Indonesia [38]. A protective effect of education on smoking, after controlling for other factors, was consistent with results of previous studies [16]-[18]. However, association of smoking with wealth index was consistent for women in most countries, but not for men, highlighting that smoking behavior may be context-specific, needing country-level analysis like that seen in the GATS report [17] but not in other reports from WHS [16] and DHS [18]. In developed countries, the smoking epidemic began among the rich and educated and later spread to lower socio-economic groups [39], but in developing countries the less educated may have taken up smoking, due to lack of awareness about health risks [40]. Lack of association of wealth index with smoking in some countries could be explained by parental influences, peer pressure [41], and cultural acceptance of smoking [42]. Significantly higher rates of smoking among urban residents have been reported [18],[43]; in our study a higher prevalence of tobacco use among rural residents was not significant for men (with multivariate analysis). We found that socio-demographic factors associated with SLT use were increasing age, lower education, and poverty among both men and women, which is similar to determinants of current smoking in our study and studies from India [26], Nepal [25], and Bangladesh [44], which analyzed SLT use separately. These findings are also similar to multicountry surveys that reported social determinants of tobacco use [17] or smoking only [16],[18].

An advantage of using DHS data was that DHS included large representative samples of men and women and allowed cross-country comparison of tobacco prevalence. Our analysis has revealed, albeit with some limitations, that comparable estimates of current tobacco use can be obtained from DHS. However, future DHS in more than 85 LMICs should include more questions about previous tobacco use and quit attempts to provide data for monitoring of the global tobacco epidemic [45],[46]. Although GATS intends to include more countries [34] in future surveys, DHS cover most LMICs [30] where the majority of tobacco-attributable deaths occur [4]. Our analysis fulfills the need for assessing the social disparities in tobacco use [24],[42] and studying the pattern of tobacco use [26] in South and Southeast Asia, which already has a high burden of communicable and nutrition-related diseases [47]. Urgent actions are necessary to reduce the burden of NCDs [48]. If nothing is done, health inequalities health among the socially disadvantaged may widen further.

There are some limitations of estimates from DHS data due to its design and the questions asked about tobacco use. First, the sample of men and women aged 15–49 years leads to under-estimation, if men and women aged >49 years had higher rates of tobacco use. Second, from the limited questions asked we could only estimate current use, unlike WHS and GATS, which have provided insights into other stages of smoking behavior such as never user and former user. Third, participants were asked to quantify tobacco use by cigarettes smoked during the last 24 hours, but other forms of smoking (bidi, pipe, hookah) and SLT use were not quantified. Fourth, the associations with social factors lack temporal relationships, as DHS were cross-sectional studies. Lastly, in conservative Asian societies it is very likely that tobacco use based on self-report may be under-reported, and DHS did not verify this by measuring urinary cotinine levels.

## Conclusions

Our study provides information about prevalence and patterns of tobacco use among men and women in South and Southeast Asian countries not covered in other multicounty surveys and confirms that tobacco use was higher among men, the less educated, and the poor, particularly those living in rural areas. Policymakers should consider the diverse forms of tobacco used and social distribution in each country to provide context-specific tobacco prevention and control strategies and target vulnerable groups. Policymakers need to consider SLT use separately in tobacco control efforts, since the economic and health effects of SLT use are different from that of smoking.

## Competing interests

The authors declare that they have no competing interests.

## Authors’ contributions

CTS was principal author who conceptualized the manuscript, performed data analysis, interpreted the results, and wrote the results and discussion sections of the manuscript. PMS assisted in conceptualization of the manuscript, wrote the background and methods sections, and revised the final drafts of the manuscript. IAM assisted in conceptualization of the manuscript, assisted in data analyses, prepared the tables and graphs, and revised the final draft of the manuscript. SS assisted in conceptualization of the manuscript, assisted in revised data analyses, prepared the revised tables and results section, and revised the initial version of the manuscript. All authors read and approved the final manuscript.

## References

[B1] Murray CJ Vos T Lozano R Naghavi M Flaxman AD Michaud C Ezzati M Shibuya K Salomon JA Abdalla S Aboyans V Abraham J Ackerman I Aggarwal R Ahn SY Ali MK Alvarado M Anderson HR Anderson LM Andrews KG Atkinson C Baddour LM Bahalim AN Barker-Collo S Barrero LH Bartels DH Basáñez MG Baxter A Bell ML Benjamin EJ Disability-adjusted life years (DALYs) for 291 diseases and injuries in 21 regions, 1990–2010: a systematic analysis for the global burden of disease study 2010 Lancet 2012 380 2197 2223 10.1016/S0140-6736(12)61689-4 23245608

[B2] Lim SS Vos T Flaxman AD Danaei G Shibuya K Adair-Rohani H Amann M Anderson HR Andrews KG Aryee M Atkinson C Bacchus LJ Bahalim AN Balakrishnan K Balmes J Barker-Collo S Baxter A Bell ML Blore JD Blyth F Bonner C Borges G Bourne R Boussinesq M Brauer M Brooks P Bruce NG Brunekreef B Bryan-Hancock C Bucello C A comparative risk assessment of burden of disease and injury attributable to 67 risk factors and risk factor clusters in 21 regions, 1990–2010: a systematic analysis for the Global Burden of Disease Study 2010 Lancet 2012 380 2224 2260 10.1016/S0140-6736(12)61766-8 23245609 PMC4156511

[B3] Detels R Beaglehole R Lansang MA Gulliford M Oxford Textbook of Public Health 2011 Oxford University Press, New York

[B4] Ezzati M Lopez AD Estimates of global mortality attributable to smoking in 2000 Lancet 2003 362 847 852 10.1016/S0140-6736(03)14338-3 13678970

[B5] Ng M Freeman MK Fleming TD Robinson M Dwyer-Lindgren L Thomson B Wollum A Sanman E Wulf S Lopez AD Murray CJ Gakidou E Smoking prevalence and cigarette consumption in 187 countries, 1980–2012 JAMA 2014 311 183 192 10.1001/jama.2013.284692 24399557

[B6] Mathers C Stevens G Mascarenhas M Global Health Risks: Mortality and Burden of Disease Attributable to Selected Major Risks 2009 World Health Organization, Geneva

[B7] WHO Report on the Global Tobacco Epidemic, 2011: Warning About the Dangers of Tobacco 2011 World Health Organization, Geneva

[B8] Sinha DN Palipudi KM Rolle I Asma S Rinchen S Tobacco use among youth and adults in member countries of South-East Asia region: review of findings from surveys under the Global Tobacco Surveillance System Indian J Public Health 2011 55 169 10.4103/0019-557X.89946 22089684

[B9] Thakur JS Garg R Narain JP Menabde N Tobacco use: a major risk factor for non communicable diseases in South-East Asia region Indian J Public Health 2011 55 155 10.4103/0019-557X.89943 22089682

[B10] Kyaing NN Islam MA Sinha DN Rinchen S Social, economic and legal dimensions of tobacco and its control in South-East Asia region Indian J Public Health 2011 55 161 10.4103/0019-557X.89944 22089683

[B11] Sinha DN Gupta PC Ray CS Singh PK Prevalence of smokeless tobacco use among adults in WHO South-East Asia Indian J Cancer 2012 49 342 10.4103/0019-509X.107726 23442396

[B12] Boffetta P Hecht S Gray N Gupta P Straif K Smokeless tobacco and cancer Lancet Oncol 2008 9 667 675 10.1016/S1470-2045(08)70173-6 18598931

[B13] Gupta PC Ray CS Smokeless tobacco and health in India and South Asia Respirology 2003 8 419 431 10.1046/j.1440-1843.2003.00507.x 14708551

[B14] Barbeau EM Leavy-Sperounis A Balbach ED Smoking, social class, and gender: what can public health learn from the tobacco industry about disparities in smoking? Tob Control 2004 13 115 120 10.1136/tc.2003.006098 15175523 PMC1747877

[B15] Jarvis MJ, Wardle J: **Social patterning of individual health behaviours: the case of cigarette smoking.** Edited by Marmot M, Wilkinson RG. Oxford: Oxford University Press; 2005.

[B16] Hosseinpoor AR Parker LA d’Espaignet ET Chatterji S Socioeconomic inequality in smoking in low-income and middle-income countries: results from the World Health Survey PLoS One 2012 7 e42843 10.1371/journal.pone.0042843 22952617 PMC3430638

[B17] Palipudi KM Gupta PC Sinha DN Andes LJ Asma S McAfee T Social determinants of health and tobacco use in thirteen low and middle income countries: evidence from Global Adult Tobacco Survey PLoS One 2012 7 e33466 10.1371/journal.pone.0033466 22438937 PMC3306395

[B18] Pampel F Tobacco use in sub-Sahara Africa: estimates from the demographic health surveys Soc Sci Med 2008 66 1772 1783 10.1016/j.socscimed.2007.12.003 18249479 PMC2679748

[B19] Mathur C Stigler MH Perry CL Arora M Reddy KS Differences in prevalence of tobacco use among Indian urban youth: the role of socioeconomic status Nicotine Tob Res 2008 10 109 116 10.1080/14622200701767779 18188751

[B20] Mackay J Amos A Women and tobacco Respirology 2003 8 123 130 10.1046/j.1440-1843.2003.00464.x 12753525

[B21] Warren CW Asma S Lee J Lea V Mackay J Global tobacco surveillance system The GTSS atlas Atlanta, Georgia, Centers for Disease Control and Prevention 2009 9 9

[B22] Bonita R De C Dwyer T Jamrozik K Winkelmann R The WHO Stepwise Approach to Surveillance (STEPS) of NCD Risk Faktors 2001 World Health Organization, Geneva

[B23] Ustün TB Chatterji S Mechbal A Murray CJ The World Health Surveys Health Systems Performance Assessment: Debates, Methods and Empiricism 2003 World Health Organization, Geneva 797

[B24] Harper S McKinnon B Global socioeconomic inequalities in tobacco use: internationally comparable estimates from the World Health Surveys Cancer Causes Control 2012 23 11 25 10.1007/s10552-012-9901-5 22350860

[B25] Sreeramareddy CT Ramakrishnareddy N Harsha KH Sathian B Arokiasamy JT Prevalence, distribution and correlates of tobacco smoking and chewing in Nepal: a secondary data analysis of Nepal Demographic and Health Survey-2006 Subst Abuse Treat Prev Policy 2011 6 33 10.1186/1747-597X-6-33 22185233 PMC3266635

[B26] Rani M Bonu S Jha P Nguyen SN Jamjoum L Tobacco use in India: prevalence and predictors of smoking and chewing in a national cross sectional household survey Tob Control 2003 12 e4 10.1136/tc.12.4.e4 14660785 PMC1747786

[B27] Subramanian SV Nandy S Kelly M Gordon D Smith GD Patterns and distribution of tobacco consumption in India: cross sectional multilevel evidence from the 1998–9 national family health survey BMJ 2004 328 801 806 10.1136/bmj.328.7443.801 15070637 PMC383376

[B28] Ansara DL Fred A Sunita K Jason H Rachel K Tobacco use by men and Women in 49 Countries With Demographic and Health Surveys: DHS Comparative Reports. 31 2014 ICF International, Calverton, Maryland, USA

[B29] Demographic and Health Surveys 2009 ICF Macro, Calverton, MD

[B30] Corsi DJ Neuman M Finlay JE Subramanian SV Demographic and health surveys: a profile Int J Epidemiol 2012 41 1602 1613 10.1093/ije/dys184 23148108

[B31] Rutstein SO Johnson K Demographic and Health Survey Wealth Index 2004 ORC Macro, MEASURE DHS, Calverton, MD

[B32] Vyas S Kumaranayake L Constructing socio-economic status indices: how to use principal components analysis Health Policy Plan 2006 21 459 468 10.1093/heapol/czl029 17030551

[B33] StataCorp LP Stata/IC 10, 0 for Windows 2007 College Station, Texas

[B34] Giovino GA Mirza SA Samet JM Gupta PC Jarvis MJ Bhala N Peto R Zatonski W Hsia J Morton J Palipudi KM Asma S Tobacco use in 3 billion individuals from 16 countries: an analysis of nationally representative cross-sectional household surveys Lancet 2012 380 668 679 10.1016/S0140-6736(12)61085-X 22901888

[B35] Singh PN Yel D Sin S Khieng S Lopez J Job J Ferry L Knutsen S Tobacco use among adults in Cambodia: evidence for a tobacco epidemic among women Bull World Health Organ 2009 87 905 912 10.2471/BLT.08.058917 20454481 PMC2789360

[B36] Toll BA Ling PM The Virginia Slims identity crisis: an inside look at tobacco industry marketing to women Tob Control 2005 14 172 180 10.1136/tc.2004.008953 15923467 PMC1748044

[B37] Sinha DN Bajracharya B Khadka BB Rinchen S Bhattad VB Singh PK Smokeless tobacco use in Nepal Indian J Cancer 2012 49 352 356 10.4103/0019-509X.107728 23442398

[B38] Webster PC Indonesia: the tobacco industry’s“Disneyland” CMAJ 2013 185 E97 E98 10.1503/cmaj.109-4342 23296586 PMC3563903

[B39] Lopez AD Collishaw NE Piha T A descriptive model of the cigarette epidemic in developed countries Tob Control 1994 3 242 10.1136/tc.3.3.242

[B40] Siahpush M McNeill A Hammond D Fong GT Socioeconomic and country variations in knowledge of health risks of tobacco smoking and toxic constituents of smoke: results from the 2002 International Tobacco Control (ITC) Four Country Survey Tob Control 2006 15 iii65 iii70 16754949 10.1136/tc.2005.013276 PMC2593062

[B41] Simons-Morton B Haynie DL Crump AD Eitel P Saylor KE Peer and parent influences on smoking and drinking among early adolescents Health Educ Behav 2001 28 95 107 10.1177/109019810102800109 11213145

[B42] David A, Esson K, Perucic AM, Fitzpatrick C: **Tobacco use: equity and social determinants.** *Equity, Social Determinants and Public Health Programmes* 2010, **199**.

[B43] Völzke H Neuhauser H Moebus S Baumert J Berger K Stang A Ellert U Werner A Döring A Urban–rural disparities in smoking behaviour in Germany BMC Public Health 2006 6 146 10.1186/1471-2458-6-146 16756650 PMC1513566

[B44] Palipudi KM Sinha DN Choudhury S Zaman MM Asma S Andes L Dube S Predictors of tobacco smoking and smokeless tobacco use among adults in Bangladesh Indian J Cancer 2012 49 387 392 10.4103/0019-509X.107745 23442403 PMC6349135

[B45] Warren CW Asma S Lee J Lea V Mackay J Global tobacco surveillance system The GTSS Atlas Atlanta, Georgia, Centers for Disease Control and Prevention 2009

[B46] Giovino GA Biener L Hartman AM Marcus SE Schooley MW Pechacek TF Vallone D Monitoring the tobacco use epidemic I. Overview: optimizing measurement to facilitate change Prev Med 2009 48 S4 S10 10.1016/j.ypmed.2008.08.007 18809429

[B47] Lozano R Naghavi M Foreman K Lim S Shibuya K Aboyans V Abraham J Adair T Aggarwal R Ahn SY Alvarado M Anderson HR Anderson LM Andrews KG Atkinson C Baddour LM Barker-Collo S Bartels DH Bell ML Benjamin EJ Bennett D Bhalla K Bikbov B Bin Abdulhak A Birbeck G Blyth F Bolliger I Boufous S Bucello C Burch M Global and regional mortality from 235 causes of death for 20 age groups in 1990 and 2010: a systematic analysis for the Global Burden of Disease Study 2010 Lancet 2012 380 2095 2128 10.1016/S0140-6736(12)61728-0 23245604 PMC10790329

[B48] Beaglehole R Bonita R Horton R Adams C Alleyne G Asaria P Baugh V Bekedam H Billo N Casswell S Cecchini M Colagiuri R Colagiuri S Collins T Ebrahim S Engelgau M Galea G Gaziano T Geneau R Haines A Hospedales J Jha P Keeling A Leeder S Lincoln P McKee M Mackay J Magnusson R Moodie R Mwatsama M Priority actions for the non-communicable disease crisis Lancet 2011 377 1438 1447 10.1016/S0140-6736(11)60393-0 21474174

